# Posterolateral approach for posterior malleolus fixation in ankle fractures: functional and radiological outcome based on Bartonicek classification

**DOI:** 10.1007/s00402-022-04620-0

**Published:** 2022-10-02

**Authors:** Lei Yang, Gang Yin, Jianguo Zhu, Haifeng Liu, Xiaoqiang Zhao, Lei Xue, Fen Yin, Jinbo Liu, Zhiyuan Liu

**Affiliations:** 1 Department of Orthopedics, Wujin Hospital Affiliated with Jiangsu University, Changzhou, 213017 China; 2 Department of Orthopedics, The Wujin Clinical College of Xuzhou Medical University, Changzhou, 213017 China; 3 Department of Spinal Surgery, The Third Affiliated Hospital of Soochow University, Changzhou, 213003 China

**Keywords:** Posterior malleolus fracture, Posterolateral approach, Bartonicek classification, Bartonicek II subtypes, Clinical outcome

## Abstract

**Introduction:**

Posterolateral approach has been advocated for the treatment of ankle fractures involving the posterior malleolus and satisfactory results were demonstrated in several studies. The Bartonicek classification based on 3-dimensional CT scanning was commonly used for treatment recommendation of posterior malleolar fracture (PMF). The aim of this retrospective study was to evaluate the clinical effect of the posterolateral approach for the treatment of PMF and present outcomes of patients with different types of Bartonicek classification.

**Method:**

We retrospectively reviewed the clinical outcomes of 72 patients with ankle fractures involving posterior malleolus (PM) from January 2016 to December 2018. Posterior malleolus fractures (PMFs) were all directly reduced and fixed by a posterolateral approach using lag screws and/or buttress plates. AOFAS score and VAS pain score were used as the primary functional outcome measures. The radiographic evaluation included the quality of the reduction and Kellgren–Lawrence (KL) osteoarthritis classification.

According to the CT-based Bartonicek classification, all patients were classified into three groups: 42 type II, 18 type III and 12 type IV. Bartonicek type II patients were further divided into subtype IIa 19 cases, subtype IIb 16 cases and subtype IIc 7 cases. The radiological and functional outcomes were analyzed among different types and subtypes of Bartonicek classification.

**Results:**

Sixty-eight patients (94.5%) achieved good or excellent reduction of PMF after surgery. The mean AOFAS score was 81.35 ± 6.15 at 6 months and 90.56 ± 4.98 at the final follow-up, respectively. The VAS score was 6.62 ± 1.03 one week after surgery, and 1.20 ± 0.92 at the final follow-up. Radiological evaluation at the final follow-up showed that primary bone union was achieved in all patients and 65 patients (88.9%) got no (KL grade 0) or just doubtable (KL grade 1) post-traumatic osteoarthritis. AOFAS scores decreased significantly with the severity of Bartonicek classification at 6 month (*p* < 0.001) and final follow-up (*p* < 0.05), while there was no statistical difference of VAS pain score among different types of Bartonicek classification. Reduction quality and the presence of osteoarthritis was not correlated to Bartonicek classification either. Besides, AOFAS scores at the final follow-up were statistically different among three subtypes of Bartonicek type II fractures (*p* < 0.05), and Bartonicek subtype IIa fractures had the highest AOFAS scores as 93 ± 4.99. Presence and severity of osteoarthritis was lower in patients with subtype IIa PMF compared to other subtype groups, this finding was statistically significant (*p* < 0.05).

**Conclusion:**

The posterolateral approach could achieve good clinical outcomes in the treatment of posterior malleolus fracture. Patients with a Bartonicek type II fracture had a better functional outcome measured by the AOFAS score compared to other types. Bartonicek type IIa fractures got a higher AOFAS score and a lower incidence of osteoarthritis at the final follow-up than the other two subtypes. Classification of PMFs according to the Bartonicek classification was reliable.

## Introduction

Ankle fractures are a relatively common type of limb fracture, while PMFs approximately account for 7–44% of all ankle fractures [1]. However, treatment of PMF remains nowadays a controversial topic. It was once widely accepted that the PM fracture requires further surgical fixation when the size of the posterior marginal fragment is more than 25% of the articular surface accompanied by displacement [2, 3]. Recently, several studies demonstrated that the presence of a PMF regardless of size has a negative influence on the outcome and should be treated properly [4, 5]. Drijfhout et al. found that medium and large sized fragments and postoperative step-off of the PM (≥ 1 mm) might increase the incidence of radiographic osteoarthritis [6]. On the other hand, anatomical reduction and fixation of PM fractures could provide better syndesmotic stability and articular surface congruity, which are associated with short-term clinical outcomes [7, 8].

Direct open reduction and fixation of posterior malleolar fragments from posterior to anterior could provide a better biomechanical stability and a more accurate reduction. Subsequently, the posterolateral approach has been advocated for the treatment of PFM and good results were demonstrated in several researches of the treating PMF by posterior approaches [9–11].

In recent years, it has become recognized that the size of the posterior malleolar fragment is not the only interfering factor of prognosis. Literatures have shifted the focus towards fracture morphology rather than fragment size [12]. The utilization of CT scan makes it possible to assess the exact shape of the posterior malleolar fragments, leading to a better understanding of the anatomical pattern of PMF. Several classification systems have been developed [13–15]. The first classification system, based on 3-dimensional (3D) CT reconstruction, was proposed by Bartonicek and Rammelt in 2015 [14]. PMFs were classified as 4 basic types with constant reference to the involvement of the fibular notch. On the basis of these characterizations of the PMF, the treatment recommendations have been proposed [16, 17].

In this study, the functional and radiological results were reviewed in 72 patients with PM ankle fracture treated using a posterolateral approach with screws and/or buttress plates. We further compared the outcomes of patients with different types of Bartonicek classification. We also found superior ankle functional recovery in Bartonicek subtype IIa PMF after surgery, to our knowledge this study was the first to analyze differences in clinical outcome according to Bartonicek type II sub-classification.

## Patients and methods

This retrospective study was conducted at a level one trauma center. Approval for the study was obtained from our Institutional Review Board.

### Patients

Patients with ankle fracture were screened to identify candidates meeting inclusion criteria of (1) ankle fracture involving the PM, (2) aged 18 years and above, (3) PMF was surgically fixed with lag screws and/or buttress plates via a posterolateral approach, (4) underwent pre- and post-operative X-rays and CT examination of the ankle, including a 3D CT reconstruction, (5) a minimum follow-up of 2 years postoperatively. Patients were excluded if they had tibia pilon fractures, open fractures, history of ankle fractures, and pathological fractures. A total of 72 patients were finally recruited between January 2016 and December 2018.

Interobserver agreement for the Bartonicek classification was determined by first and senior authors (Liu Zhiyuan and Yang lei). Disagreements between observers were resolved by an extra discussion.

**Fig. 1:**
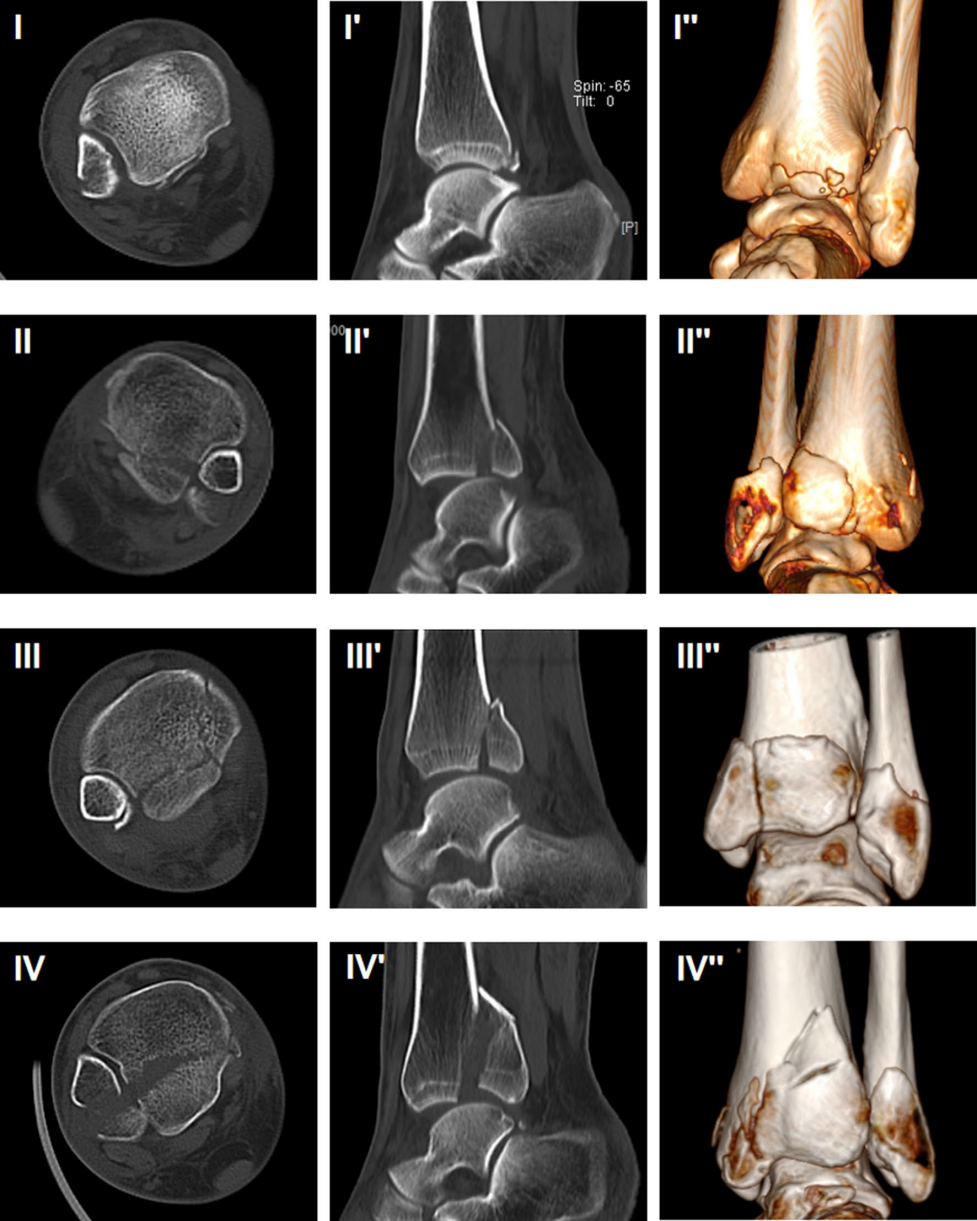
Bartonicek classification of PMF. (I, I′, II″) Extraincisural fragment. (II, II′, II″) Posterolateral fragment. (III, III′, III″) Posteromedical, two-part fragment. (IV, IV′, IV″) Large, posterolateral triangular fragment

These patients were divided into three groups based on the morphology of PM according to Bartonicek classification (Fig. 1):

- *Type II*: intraincisural posterolateral fragment involving the fibular notch.
- *Type III*: intraincisural posteromedial two-part fragment involving the posterior part of the fibular notch, and the posterior colliculus of the medial malleolus.
- *Type IV*: large posterolateral triangular fragment carrying the posterior half of the fibular notch.

**Fig. 2:**
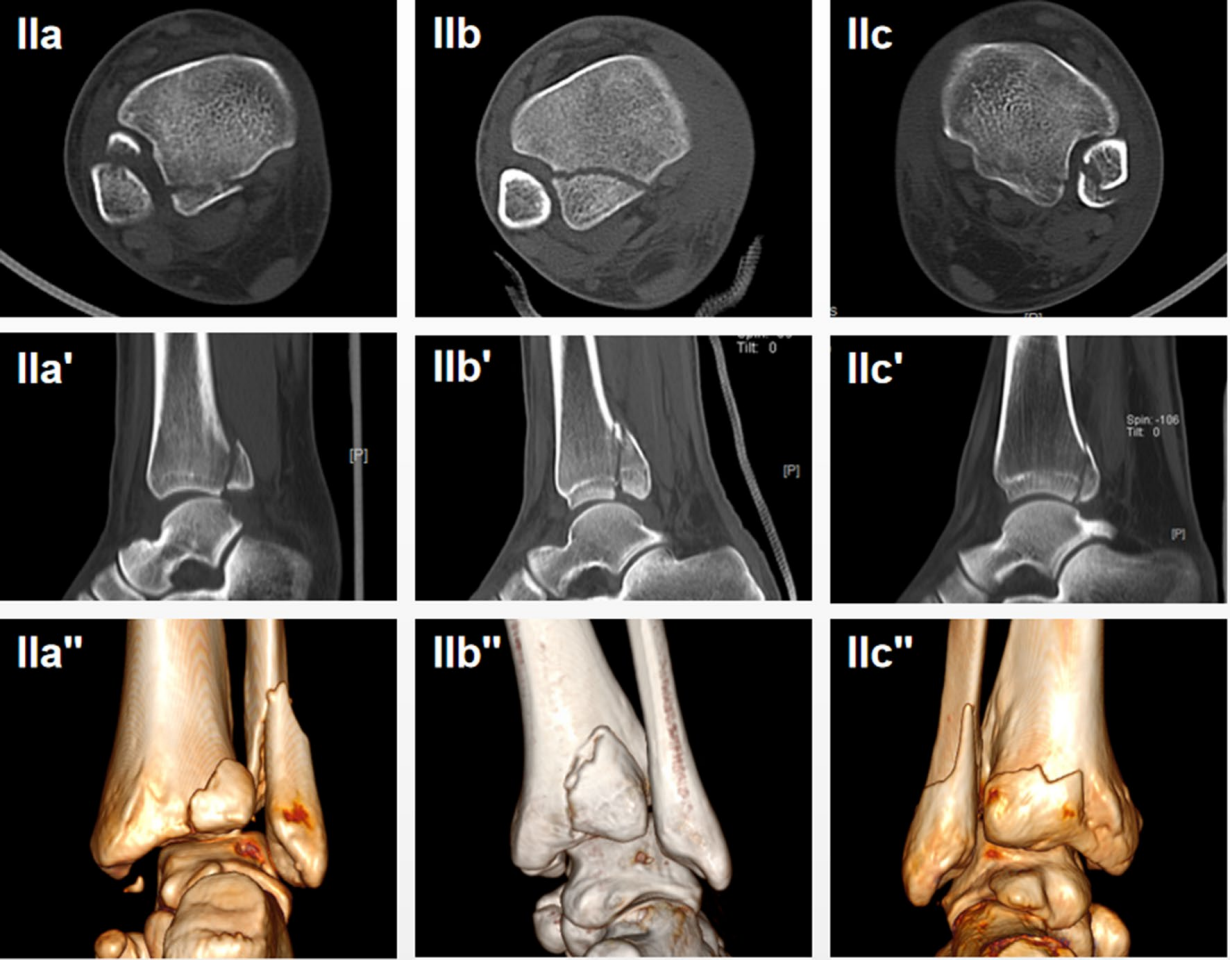
Variability of Bartonicek classification type II PMF (intraincisural posterolateral fragment) on CT scans in horizontal plane (IIa, IIb, IIc), sagittal plane (IIa′, IIb′, IIc′) and 3D reconstruction (IIa″, IIb″, IIc″)

Variable Type II PMF were further divided into three subtype groups as (Fig. 2):

- *Type IIa*: a small fragment involving the fibular notch.
- *Type IIb*: typical fragment involving 1/4–1/3 the fibular notch.
- *Type IIc*: a integrated larger fragment including thin layer medial extension fragment.

Demographic characteristics, injury mechanism, fracture type including Bartonicek classification, and operative characteristics are shown in Table 1.

**Table 1:** Patient, fracture and operative characteristics

Variable	Values
Age (y)	21–83 (52.0 ± 16.2)
Gender	
Male	26 (36.1%)
Female	46 (63.9%)
Injury mechanism	
Fall	9 (12.5%)
Twist/slide	34 (47.2%)
Cycling injury	13 (18.1%)
Accident	16 (22.2%)
Localization	
Left	30 (41.7%)
Right	42 (58.3%)
Fracture type	
Posterior	1 (1.4%)
Posterior and fibula	5 (6.9%)
Posterior and medial	4 (5.6%)
Trimalleolar	62 (86.1%)
Fracture dislocation	
Yes	42 (58.3%)
No	30 (41.7%)
Bartoníček/Rammelt classification	
Type I	0 (0%)
Type II	42 (58.3%)
IIa	19 (45.2%)
IIb	16 (38.1%)
IIc	7 (16.7%)
Type III	18 (25%)
Type IV	12 (16.7%)
Posterior malleolus fixation	
PA lag screws	7 (65.3%)
Buttress plate with/without PA lag screws	25 (34.7%)
Syndesmotic fixation	
Yes	30 (41.7%)
Screw	26 (86.7%)
Endobutton	4 (13.3%)
No	42 (58.3%)

### Operative procedure and rehabilitation

All procedures were performed with the use of tourniquet and under fluoroscopy. Patients were either in the prone or lateral position. A posterolateral approach was performed through the interval between the peroneal tendons and flexor hallucis longus to gain access to the posterior malleolus. Care is taken to avoid injury to the sural nerve, flexor hallucis longus, and the posterior inferior tibiofibular ligament. When clearly exposed, the PM was reduced directly and held temporarily with K wires or a pointed reduction clamp. After satisfactory fracture reduction was confirmed, PM was fixed with posteroanterior lag screws and/or buttress plates (Small T plate or 1/3 tubular plate).

The fibular fracture was reduced and just temporarily fixed. After the treatment of PMF was finished, fibular osteosynthesis was performed using anatomical or 1/3 tubular plate. Medial malleolus fracture was treated after PMF in supine position with lag screws. Finally, an additional syndesmotic screw or endobutton system was applied when instability of the distal tibiofibular syndesmosis still existed.

The postoperative treatment of ankle fractures was to allow a range of motion and stretching exercise after surgery. Non-weight bearing continued for at least six weeks until there was radiographic evidence of fracture healing. All patients were instructed to begin weight-bearing at 6 weeks aiming for full weight-bearing by 12 weeks. The syndesmotic screw was removed before full weight-bearing.

### Outcome evaluation

Patients were evaluated by functional examination and radiography.

The quality of reduction of all fractures was assessed using postoperative CT scan according to articular step-off and/or surface gap.

The reduction was considered excellent (< 1 mm), good (1–2 mm), and poor (> 2 mm) as proposed by Ketz [18]. Loss of reduction and bone union rate were also analyzed. Osteoarthritis (OA) grade of the ankle joint at the last follow-up was performed using the Kellgren and Lawrence (KL) classification [19]:

- Grade 0—normal, no changes;
- Grade1—doubtable, possible narrowing of joint space and osteophytic lipping;
- Grade 2—mild, definite osteophytes and possible narrowing of joint space;
- Grade 3—moderate, multiple osteophytes, definite narrowing of joint space, small pseudocystic areas and possible deformity of bone contour;
- Grade 4—severe, large osteophytes, marked narrowing of joint space, severe sclerosis and definite deformity of bone contour.

The functional outcomes of all patients were evaluated primarily using the American Orthopaedic Foot and Ankle Society (AOFAS) ankle-hindfoot scores 6 months after surgery and at the final follow-up. The AOFAS consists of three parts (pain, function and alignment), where subjective and objective measures make up a scale from 0 to 100 [20]. The Visual Analog Scale (VAS) was used to quantify pain one week after surgery, and at the final follow-up.

### Statistical analysis

SPSS version 19.0 (SPSS Inc. Chicago, Illinois, USA) and GraphPad Prism, version 7.0 (GraphPad, Inc., San Diego, CA) were used for statistical analysis.

Continuous variables were presented as means and standard deviations. Categorical variables were represented by absolute and relative frequencies.

Kolmogorov–Smirnov test was used to assess normal distribution characteristics of AOFAS/VAS score. A one-way ANOVA test or Kruskal–Wallis test was used to compare the AOFAS /VAS scores of different types and subtypes of Bartonicek classification, followed by Tukey's or Dunn's multiple comparisons test. Association between the degree of injury according to Bartonicek classification and reduction quality or severity of osteoarthritis was investigated with Fisher's test.

A *p* value < 0.05 was considered statistically significant.

## Results

Basic demographic data, fracture and operative characteristics are reported in Table 1. In 42 out of 72 patients (58.3%), the trauma also caused an ankle fracture-

dislocation. Additional syndesmosis screws (26 cases) or endobutton system (4 cases) was used in 41.7% of the patients after fracture fixation.

### Radiological results

Sagittal CT scans after surgery revealed excellent reduction with a congruent ankle joint in 48 cases (66.7%), 20 cases had good reduction (27.8%), while 4 (5.5%) cases had poor reduction (Table 2). All fractures involving the fibula, PM and medial malleolus, healed within 3 months postoperatively. No invalidation or breakage of internal fixation had occurred. At the final follow-up, 65 patients (90.3%) got no (KL grade 0) or just doubtable (KL grade 1) post-traumatic osteoarthritis. 7 patients developed mild or moderate osteoarthritic changes.

**Table 2:** Radiological and functional outcome evaluation

Quality of reduction	
Excellent	48 (66.7%)
Good	20 (27.8%)
Poor	4 (5.5%)
VAS scores	
1 Week	3.90 ± 0.61
At the final follow-up	1.74 ± 0.69
AOFAS score	
6 Month	81.35 ± 6.15
At the final follow-up	90.56 ± 4.98
Kellgren–Lawrence (KL) osteoarthritis classification	
Grade 0	34 (47.2%)
Grade 1	31 (43.1%)
Grade 2	5 (6.9%)
Grade 3	2 (2.8%)
Grade 4	0

### Functional results

The mean AOFAS score for the evaluation of ankle function was 81.35 ± 6.15 at 6 months and 90.56 ± 4.98 at the final follow-up, respectively. The VAS score was 6.62 ± 1.03 one week after surgery, and 1.20 ± 0.92 at the final follow-up (Table 2).

### Comparison of clinical results according to Bartonicek classification

All 72 patients were classified according to Bartonicek classification: We found 42 (58.3%) type II, 18 (25%) type III and 12 (16.7%) type IV fractures.

No differences in patient demographics or fracture characteristics were found among three different groups (*p* > 0.05), as summarized in Table 3.

**Table 3:** Demographic data, fracture and surgical characteristics, clinical and radiological results according to Bartoníček/Rammelt (B/R) classification

	B/R II (*n* = 42)	B/R III (*n* = 18)	B/R IV (*n* = 12)	*p*
Age	50.55 ± 16.05	53.17 ± 18.36	55.17 ± 13.97	0.65
Gender				
Male	16 (38.1%)	8 (44.4%)	2 (16.7%)	0.20
Female	26 (61.9%)	10 (55.6%)	10 (83.3%)	
Localization				
Left	21 (50.0%)	3 (16.7%)	6 (50.0%)	0.27
Right	21 (50.0%)	15 (83.3%)	6 (50.0%)	
Injury mechanism				
Fall	7 (16.7%)	0	2 (16.7%)	
Twist/slide	21 (50.0%)	8 (44.4%)	3 (25.0%)	
Cycling injury	6 (14.3%)	7 (38.9%)	2 (16.7%)	0.05
Accident	8 (19.0%)	3 (16.7%)	5 (41.6%)	
Fracture dislocation				
Yes	25 (59.5%)	7 (38.9%)	10 (83.3%)	0.27
No	17 (40.5%)	11 (61.1%)	2 (16.7%)	
Syndesmotic fixation				
Yes	21 (50.0%)	4 (22.2%)	5 (41.7%)	0.18
No	21 (50.0%)	14 (77.8%)	7 (58.3%)	
Quality of reduction				
Excellent	29 (69.0%)	12 (66.7%)	7 (58.3%)	
Good	11 (26.2%)	5 (27.8%)	4 (33.3%)	0.28
Poor	2 (4.8%)	1 (5.5%)	1 (8.3%)	
AOFAS score				
6 month	85.29 ± 4.92	81.56 ± 5.10	76.42 ± 6.13	< 0.001*
At the final follow-up	91.74 ± 4.84	89.33 ± 4.77	88.25 ± 4.88	0.0286*
VAS scores				
1 week	3.81 ± 0.59	4.06 ± 0.64	4.00 ± 0.60	0.29
At the final follow-up	1.71 ± 0.71	1.83 ± 0.62	1.67 ± 0.78	0.68
K–L osteoarthritis classification				
Grade 0	21 (50.0%)	9 (50.0%)	4 (33.3%)	
Grade 1	19 (45.2%)	6 (33.3%)	6 (50.0%)	
Grade 2	1 (2.4%)	2 (11.1%)	2 (16.7%)	0.25
Grade 3	1 (2.4%)	1 (5.6%)	0	
Grade 4	0	0	0	

A one-way ANOVA test or Kruskal–Wallis test was used to evaluate the difference of functional scores between the various groups at different follow-up times. The mean AOFAS scores decreased with the severity of Bartonicek classification both at 6 month (*p* < 0.001) and at the final follow-up (*p* < 0.05). Bartonicek type II fractures had the highest AOFAS score at 6 month (85.29 ± 4.92) and the final follow-up (91.74 ± 4.84). There were no statistically significant differences of VAS scores between the various groups at 1 week (*p* = 0.29) and final follow-up (*p* = 0.68).

The Fisher's test was used to investigate the possible relationship between radiological results and Bartonicek classification. The p-values for quality of reduction were 0.28, for K–L osteoarthritis grade 0.25, showing no significant association between these variables (Table 3).

### Comparison of clinical results among Bartonicek II subtypes

To analyze potential influence of variant intraincisural PM fragment on clinical results, Type II fractures were further divided into 3 subtype groups (group IIa = 19, group IIb = 16, group IIc = 7), as described above.

**Fig. 3:**
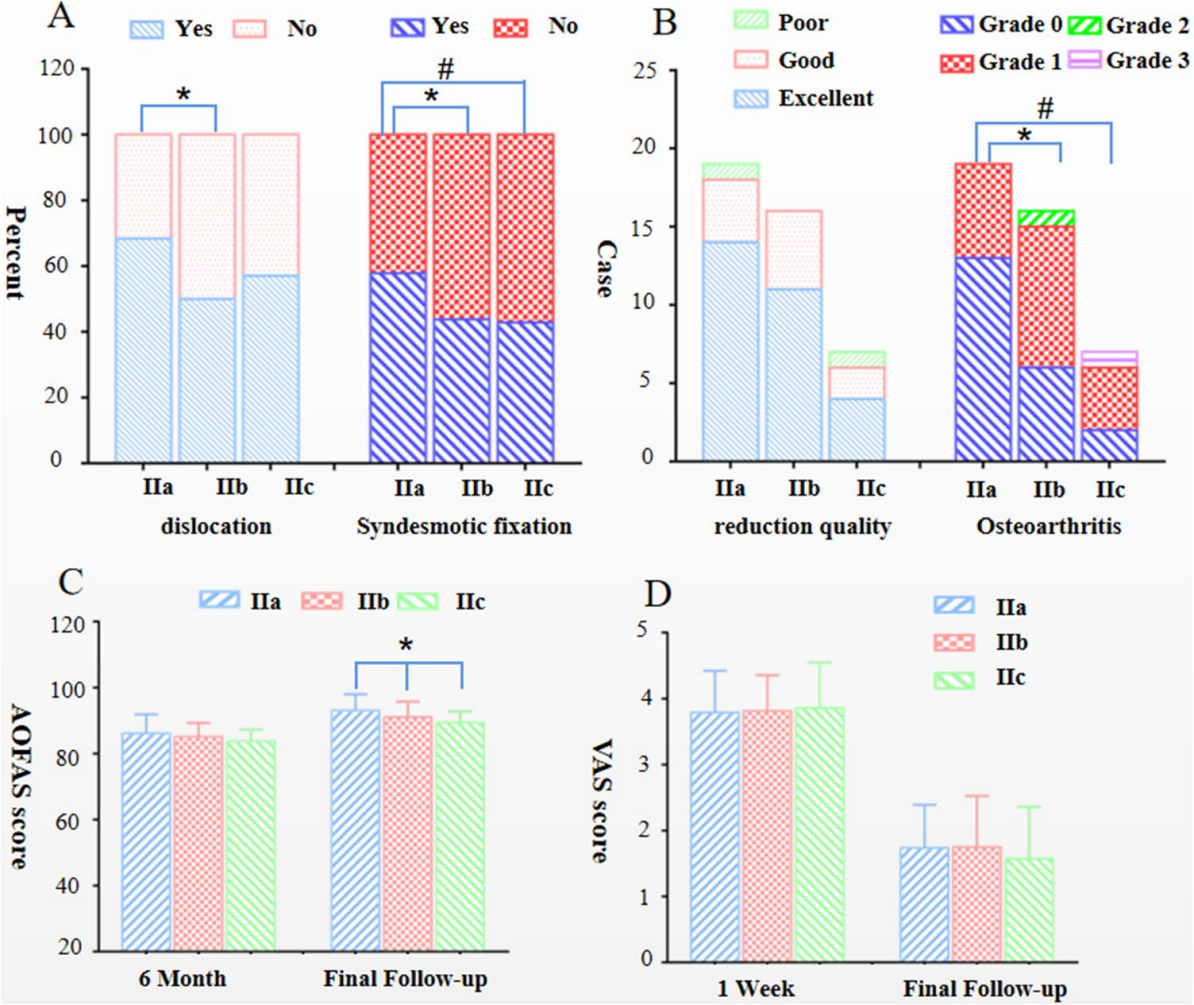
Fracture and surgical characteristics, clinical results among different subtypes of Bartonicek type II fractures

Occurrence of fracture-dislocation was not statistically different among three groups (*p* = 0.07), although group IIa was more likely to be accompanied by ankle dislocation than group IIb (*p* < 0.05). The proportion of patients requiring additional syndesmotic fixation after osteosynthesis in Group IIa (57.9%) was higher than that in Group IIb (43.8%) and group IIc (42.9%), as shown in Fig. 3A.

More patients obtained excellent reduction of PMF in Group IIa (73.7%) than the other two groups, however, there was no statistical significance of quality of reduction among different Bartonicek subtype groups (*p* = 0.26). On the other hand, radiographs taken at the final follow-up revealed more patients with lower grade of osteoarthritis in group IIa than in group IIb (*p* < 0.05) or group IIc (*p* < 0.05) (Fig. 3B).

The mean AOFAS score was gradually dropped with the increased involvement of the PM. There was no difference between the three groups at 6 months by one-way ANOVA test (*p* = 0.26), but the final AOFAS scores were statistically different using Kruskal–Wallis test (*p* < 0.05). Bartonicek subtype IIa fractures had the highest AOFAS scores as 93 ± 4.99. However, it's worth noting that Dunn's multiple comparisons test revealed no difference of the AOFAS scores between the various groups (Fig. 3C).

VAS scores between different subtype groups were investigated with the Kruskal–Wallis test: p-value for 1 week after surgery was 0.97, for final follow-up 0.80, both showing no significant difference, as shown in Fig. 3D.

## Discussion

Posterior malleolus fractures refer to ankle fractures of the posterior articular surface of the distal tibia, which is believed to participate in the symmetrical coordination of the joint [21] and displaced PMFs often lead to a worse prognosis [22–25]. In recent years, increased attention has been paid to the importance of anatomical reduction and internal fixation of the posterior malleolus [16].

The posterolateral approach is one of the most commonly used surgical approaches for the treatment of PMFs and several literatures reported good clinical results and high patient satisfaction after surgery [26–29]. It is especially helpful with relatively small fragments and in the case of additional fragments that can’t be indirectly reduced [30, 31]. In this study, we confirmed that the posterolateral approach has the advantages of adequate exposure and reduction, and precise fixation. 94.5% of the patients reported a good or excellent reduction and no failure of fixation were observed. Besides, this approach could be a useful technique for PMF with satisfactory functional recovery and imperceptible pain, based on the AOFAS and VAS scores at the final follow-up.

The posterior malleolus is an important attachment for the posterior inferior tibiofibular ligament (PITFL). Baumbach et al. found that ORIF of posterior malleolus fragments, independent of their size, significantly reduced the frequency of trans-syndesmotic fixation compared to CRIF or untreated PMF in patients with trimalleolar ankle fractures [32]. Another retrospective review by Mengnai et al. revealed that direct posterior lateral plate fixation of PMFs, in supination external rotation type IV (SER IV) ankle fractures, could effectively stabilize the syndesmosis as well [33].Recently, Caglar et al. suggested that patients may not need additional trans-syndesmotic screw fixation after anatomic reduction of the PM and posterior inferior tibiofibular ligament complex [34]. However, in our study, additional syndesmosis screws or endobutton system was used in 41.7% patients. This phenomenon may be partly attributed to the fact that we used more posterior lag-screws (65.3%) than buttress plate (34.7%) in our PMF fixation. Moreover, traditional estimates of posterior stability may not be adequate to determine the need for posterior malleolar fixation [35]. Finally, low anatomic reduction rate (66.7%) of the posterior malleolus was also another interfering factor.

Posttraumatic osteoarthritis is seen clinically or radiographically in about one-third of patients with a PMF [36]. Several clinical studies identified osteoarthritis as a potential risk factor related to poor long-term prognosis. In a clinical retrospective study,the development of early-onset osteoarthritis contributes to functional limitation in young adults with ankle fractures involving the PM [37]. Verhage et al. found correction of intra-articular step-off of posterior malleolar fragments reduced the risk of developing osteoarthritis and, consequently, worse functional outcome after long-term follow-up [38]. Our present study demonstrated that 65 patients (88.9%) got no or just doubtable post-traumatic osteoarthritis according to Kellgren and Lawrence classification[19], which is likely to benefit from accurate PMF reduction, and subsequently resulted in satisfactory functional recovery at final follow-up.

Bartonicek et al. proposed 4 typical types of PMFs on the basis of an analysis of the 3-dimensional CT scans of 141 patients, representing a scale of increased injury severity and ankle instability [14]. The value of Bartonicek classification system is in its guidance of surgical treatment. Multiple studies have reported clinical results of ankle fractures including PFM in the light of Bartonicek classification. When evaluating 46 patients with PMF conservatively treated, clinical outcomes including OMAS/AOFAS scores worsened with the severity of Bartonicek classification, but early post-traumatic arthritis were not correlated to the injury pattern [39]. Michal et al. demonstrated good mid-term results of type IV Bartonicek classification PMFs after performing direct reduction and fixation via posterolateral or posteromedial approaches [40]. In contrast, Maarten et al. compared the functional outcome and general health status between patients who underwent plate osteosynthesis or not. They suggested that there is no indication for routine plate osteosynthesis of all types of posterior malleolus fractures [41].

To our knowledge, this is the first trial exclusively analyzing the clinical results of patients with PMFs treated by posterolateral approach with respect to Bartonicek classification. In our study, the severity of Bartonicek classification led to lower mean AOFAS scores both at 6 month and at the final follow-up. Bartonicek type II fractures had the highest AOFAS score. However, VAS scores were not statistically different between the various groups at different time points. We also revealed that post-traumatic osteoarthritis was not affected by Bartonicek classification.

Anatomical reduction of the PM fragment to restore the articular surface and the normal anatomy of the inferior tibiofibular joint has been advocated [14]. Until recently, only a few authors had studied the effect of fracture pattern on reduction of the PMFs. Stephen found that more anatomical reduction was achieved as the size of the PM fragment increased [42]. Michal et al. first assessed the reduction accuracy of Bartonicek type IV PMFs with the use of postoperative CT scans. Reduction of the PMF was assessed as anatomical in 14 cases (73.7%) and as satisfactory in five (26.3%) cases [40]. In our study, 58.3% Bartonicek type IV PMFs achieved excellent reduction quality, and 33.3% cases got good reduction. We also observed that there was no significant difference in reduction quality among three Bartonicek types, indicating that fracture morphology might not be the only factor affecting reduction of posterior malleolus.

Bartonicek type II PMF refers to an intraincisural posterolateral fragment involving 1/4–1/3 of the fibular notch and accounting for at least half of all posterior ankle fractures [13–15]. No study has so far focused on subtypes of Type II PMFs according to the Bartonicek classification. In this study, we retrospectively analyzed the fracture and surgical characteristics, clinical results among different subtypes of Bartonicek type II fractures. First, we found that subtype IIa fractures presented a higher incidence (68.5%) of ankle dislocation. Moreover, a higher proportion of subtype IIa PMFs requires additional syndesmosis fixation, compared with subtype IIb and IIc groups. These discrepancies were not consistent with traditional findings and might be attributed to injury mechanism or sample shortage. While we did not reveal significant differences in reduction quality among different subtypes of type II PMFs, we found that, for osteoarthritis, the results of type IIa were better than those of type IIb and IIc. We attributed these variable results to the fact that, compared with type IIa PMFs, type IIb or IIc fractures usually involved larger size of the articular surface extending to the medial posterior rim or depressed intercalary joint fragments [16]. Finally, the difference in AOFAS among three groups at 6 months was not of statistical significance and at the final follow-up was small. Multiple comparisons found no difference of the AOFAS scores between the various groups. We also noted that the level of pain was not significantly different either. These findings reflect the fact that subtypes of Bartonicek type II PMF may just represent a scale of anatomical increased discrepancy fragments, and have no clear association with functional results.

There are several inherent weaknesses in this study. First, it was a retrospective data collection without patients treated conservatively in our cohort. Second, surgery was conducted by different surgeons varying in their experience. It should be noted that the final outcomes might be influenced by their selection of internal fixation and judgment of syndesmotic fixation necessity. Furthermore, our subtype identification mainly was on the basis of coronal CT scan without sagittal features. Finally, despite an innovative attempt to analyze the differences in prognosis between different subtypes of Bartonicek type II PMFs, our patient cohort was relatively small and the cases in each group varied greatly. All of these may affect the reliability of our clinical data analysis.

## Conclusion

This study demonstrates that 72 patients treated for ankle fractures involving PMFs, with different Bartonicek classification, achieved good clinical outcomes through the posterolateral approach. We confirmed the relationship between the severity of PM involvement in ankle fractures on the basis of Bartonicek classification and the worsening of clinical outcomes measured by the AOFAS score. Compared with type IIb and IIc fractures, patients with Bartonicek type IIa fractures got a slightly higher AOFAS score and a remarkably lower incidence of osteoarthritis at the final follow-up. Classification of posterior malleolus fractures according to the Bartonicek classification was reliable. We would recommend all ankle fractures involving the posterior malleolus undergo preoperative CT scanning to guide treatment planning.

Direct reduction and fixation from the posterolateral approach was an effective option in Bartonicek type II, III and IV fractures. Bartonicek subtype IIb, IIc PMFs should pay more attention to achieve satisfactory clinical prognosis.
